# Risks of All‐Cause Mortality in Adults With Chronic Kidney Disease With Sarcopenia or Obesity: A Population‐Based Study

**DOI:** 10.1002/jcsm.13828

**Published:** 2025-06-05

**Authors:** Jin'e Li, Hua Tu, Yuying Zhang, Shiqi Yang, Peng Yu, Jianping Liu

**Affiliations:** ^1^ Department of Endocrinology and Metabolism Second Affiliated Hospital, Jiangxi Medical College, Nanchang University Nanchang Jiangxi China; ^2^ Jiangxi Medical College Nanchang University Nanchang Jiangxi China; ^3^ Institute for the Study of Endocrinology and Metabolism in Jiangxi Province Nanchang Jiangxi China; ^4^ Jiangxi Key Laboratory of Molecular Medicine Second Affiliated Hospital of Nanchang University Nanchang China

**Keywords:** all‐cause mortality, chronic kidney disease, fat mass, sarcopenia

## Abstract

**Background:**

The relationship between obesity, sarcopenic obesity and all‐cause mortality in chronic kidney disease (CKD) patients remains controversial. This study aims to investigate the role of low muscle mass and fat mass in the risk of all‐cause mortality in CKD patients in the United States.

**Methods:**

This study utilized data from the National Health and Nutrition Examination Survey (NHANES) conducted between 1999–2006 and 2011–2018, including 1553 adults with CKD. Multivariable Cox proportional hazards models were constructed to explore the relationship between sarcopenia, fat mass and all‐cause mortality, with nonlinear relationships assessed using restricted cubic splines. Subgroup analyses were conducted based on sex, CKD stages and the presence of sarcopenia.

**Results:**

The average age of participants was 58.15 ± 18.48 years, with 45% being male. Sarcopenia was more common in men, non‐diabetic individuals and those with lower education levels. During a median follow‐up of 119.5 months, 693 deaths from all causes were recorded. After adjusting for multiple variables, sarcopenia was significantly associated with increased all‐cause mortality risk in CKD patients (HR 1.21; 95% CI 1.00–1.45, *p* = 0.047). Participants were categorized based on body mass index (BMI) into normal (reference), sarcopenia only, obesity only and sarcopenic obesity groups. Results showed that obesity alone had a protective effect in CKD Stages I–II (HR 0.45, 95% CI 0.28–0.72, *p* = 0.001) whereas it had an opposite effect in CKD Stages III–V (CKD III: HR 1.67, 95% CI 1.07–2.60, *p* = 0.024; CKD IV–V: HR 17.51, 95% CI 1.29–238.01, *p* = 0.032). Further analysis of fat mass data revealed that compared with the lowest quartile (Q1), middle and higher quartiles of fat mass showed significant benefits in male participants (Q2: HR 0.71, 95% CI 0.51–0.99, *p* = 0.046; Q3: HR 0.62, 95% CI 0.41–0.92, *p* = 0.019) and those in CKD Stage III (Q2: HR 0.64, 95% CI 0.47–0.88, *p* = 0.006; Q3: HR 0.62, 95% CI 0.41–0.93, *p* = 0.021).

**Conclusions:**

In this longitudinal cohort study, we found that sarcopenia was associated with an increased risk of all‐cause mortality in CKD patients, whereas moderate or higher fat mass might mitigate this risk, particularly in male patients. Prognostic management for CKD patients should focus on increasing muscle mass rather than simply reducing body weight.

## 1 Introduction

Sarcopenia is characterized by the accelerated and progressive loss of skeletal muscle mass and strength, and it is highly prevalent among patients with chronic kidney disease (CKD). As CKD advances, the risk of developing sarcopenia significantly rises. This condition leads to an increased risk of adverse outcomes, including poor quality of life, frailty and mortality [1, 2, 3, 4, 5, 6]. Beyond the common mechanisms associated with age‐related sarcopenia, CKD patients face additional specific risks such as metabolic acidosis, accumulation of uremic toxins, overexpression of angiotensin II and protein‐energy malnutrition [7, 8, 9, 10, 11].

Sarcopenic obesity (SO) is characterized by the decline in muscle mass and function along with an increase in adipose tissue [12, 13]. Due to severe health consequences, including impacts on mortality, comorbidities and the risk of geriatric syndromes, SO is increasingly a concern among the elderly [14, 15, 16]. Studies have reported that SO is a significant risk factor for heart failure in patients with type 2 diabetes, and these patients can benefit from simultaneously controlling obesity and muscle strength [17]. Growing evidence suggests that both obesity and sarcopenia independently increase the risk of cardiovascular disease (CVD) [18, 19]. This has led to the expectation that elderly individuals with SO have a high risk of CVD. Some studies have put forth the counterintuitive view that the presence of obesity might mitigate the negative effects of sarcopenia [20, 21]. Therefore, whether sarcopenia or even SO increases the risk of cardiovascular events and all‐cause mortality in CKD patients remains controversial.

This study aims to utilize the National Health and Nutrition Examination Survey (NHANES) database to explore the relationship between low muscle mass, fat mass, and all‐cause mortality in the CKD population. The study seeks to provide insights for clinical practice and guide management strategies for CKD patients to improve muscle mass and control weight, ultimately enhancing patient outcomes.

## 2 Methods

### 2.1 Data Collection and Study Population

The NHANES is a biannual survey conducted by the National Center for Health Statistics (NCHS). The NCHS Ethics Review Board approved the entire programme, and all participants signed informed consent forms. All detailed NHANES study designs and data are available online (www.cdc.gov/nchs/nhanes/). This study followed the Strengthening the Reporting of Observational Studies in Epidemiology (STROBE) reporting guideline. We obtained NHANES data from 1999–2006 and 2011–2018, which included 80 630 participants. Among them, 34 181 participants were under 18 years old and were excluded. We further screened for CKD patients with estimated glomerular filtration rate (eGFR) < 60 mL/min/1.73 m^2^ or urine albumin‐to‐creatinine ratio (UACR) ≥ 30 mg/g. Additionally, we excluded patients with malignant tumours, liver cirrhosis, or those without complete dual‐energy X‐ray absorptiometry (DXA) examinations, clinical or biochemical data. Ultimately, 1553 participants were included.

### 2.2 Assessment of CKD

eGFR was estimated by Chronic Kidney Disease Epidemiology Collaboration (CKD‐EPI) 2021 equation [22]. CKD was defined as eGFR < 60 mL/min/1.73 m^2^ or UACR ≥ 30 mg/g. CKD stages are classified based on eGFR levels as I, II, IIIa, IIIb, IV and V.

### 2.3 Assessment of the Sarcopenia and Fat Mass Quartiles

In the NHANES, DXA was used to assess the body composition. Among them, appendicular lean mass (ALM) was determined as the sum of four limbs' muscle mass. Sarcopenia was defined following the Foundation for the National Institutes of Health (FNIH) definition using the ALM and body mass index (BMI) ratio [23]. The cut‐off values for sarcopenia index were < 0.789 kg/m^2^ for men and < 0.512 kg/m^2^ for women. Evaluation was based on fat mass measured by DXA, and participants were grouped into quartiles (Q1, Q2, Q3 and Q4).

### 2.4 Covariates

Based on a literature review, we summarized potential confounders that might bias the association between sarcopenia and mortality in our multivariable‐adjusted models. In our study, the covariates included gender (male/female), age, race (Mexican American/other Hispanic/non‐Hispanic White/non‐Hispanic Black/other races), education level (high school and less than high school/above high school/others), BMI, blood pressure, low‐density lipoprotein cholesterol (LDL‐C), high‐density lipoprotein cholesterol (HDL‐C), total cholesterol (TC), triglycerides (TG), eGFR, uric acid (UA), albumin, serum potassium and serum calcium. In addition, smoking status was categorized into three groups (never smoked, former smoker and current smoker). Drinking frequency was categorized into four groups (non‐drinker, 1–5 drinks/month, 5–10drinks/month and 10+ drinks/month).

Participants were considered to have diabetes if they met at least one of the following criteria: (1) diagnosis of diabetes, (2) use of hypoglycaemic medications, (3) haemoglobin A1c (HbA1C)level of 6.5%, (4) fasting plasma glucose level of 126 mg/dL or higher or (5) 2‐h plasma glucose level of 200 mg/dL or higher [24]. We used BMI data to classify the CKD participants into non‐obesity (< 28 kg/m^2^) and obesity (≥ 28 kg/m^2^) groups. All detailed measurement processes of the study variables are publicly available on the NHANES website.

Physical activity (PA) data were obtained from self‐reported questionnaires in NHANES. We calculated the total weekly metabolic equivalent minutes (MET‐min/week) based on activity type, frequency, and duration using the formula: PA = MET × weekly frequency × duration per session, applying the recommended MET values provided by NHANES. According to the US Physical Activity Guidelines, participants were categorized into three groups: inactive (< 600 MET‐min/week), active (600–1200 MET‐min/week) and highly active (≥ 1200 MET‐min/week).

The Controlling Nutritional Status (CONUT) score is calculated based on the sum of the serum albumin score, total lymphocyte count score and total cholesterol score. CONUT grouping is determined using clinically significant cut‐off values reported in previous studies, classifying participants into a low CONUT group (< 3 points) and a high CONUT group (≥ 3 points), with the high CONUT group being at a higher risk of malnutrition [25]. Daily energy intake (kcal/day) in this study was analysed based on the average energy intake from 2 days of food consumption for each participant.

### 2.5 Statistical Analyses

All analyses were conducted using R software (R Foundation for Statistical Computing, Vienna, Austria). Continuous variables are presented as the mean ± standard deviation, and categorical variables are presented as proportions. We used unadjusted and multivariable proportional hazards (Cox) regression to evaluate the linear relationship between sarcopenia and mortality in CKD patients: Model 1 (unadjusted model), Model 2 (adjusted for gender, age, race, education, BMI, history of smoking and drinking and diabetes) and Model 3 (adjusted for gender, age, race, education, BMI, smoking status, drinking frequency, diabetes, MET levels, CONUT group, diary energy intake, systolic blood pressure, serum potassium, serum calcium, serum albumin, lipid metabolism indicators [TC, TG, LDL, HDL] and eGFR levels). The effects of sarcopenia on overall mortality were evaluated with the use of Kaplan–Meier curves. Subgroup analyses were conducted based on gender, age, race, education level, smoking status, drinking frequency, diabetes, obesity, MET levels, CONUT group and CKD stage. To investigate the relationship between the sarcopenia index and all‐cause mortality, we used Cox proportional hazards regression modelling with restricted cubic splines (RCS) and smooth curve fitting (penalized spline method) with four knots. Statistical significance was set at *p* < 0.05.

## 3 Results

### 3.1 Baseline Characteristics

The mean age of the 1553 NHANES patients meeting the inclusion criteria was 58.15 ± 18.48 years, with 697 (45%) being male. Over 50% of the participants had an education level higher than high school. A total of 765 participants had a history of smoking, and 1036 had a history of alcohol consumption. Thirty percent of the participants had a history of diabetes. Based on the sarcopenia index, 23.3% (*n* = 362) of participants were considered to have clinically significant sarcopenia. The majority of adults with hypomuscular disorder were non‐Hispanic white (173 cases, 48%) and Mexican American (114 cases, 31%). Most patients with sarcopenia were male, elderly, non‐diabetic and inactive. CKD patients with sarcopenia had higher BMI, lower eGFR and higher systolic blood pressure. The overall median follow‐up was 119.5 months, during which 693 all‐cause deaths were observed. The baseline characteristics of the study participants in different groups are shown in Table 1.

**TABLE 1:** Baseline characteristics of the study population.

Characteristics	Overall, *N* = 1553^a^	Sarcopenia		*p* ^b^
		No, *N* = 1191^a^	Yes, *N* = 362^a^	
Age (years)	58.15 (18.48)	55.73 (18.67)	66.11 (15.37)	< 0.001
Male	697 (45%)	503 (42%)	194 (54%)	< 0.001
Race				< 0.001
Mexican American	302 (19%)	188 (16%)	114 (31%)	
Non‐Hispanic Black	78 (5.0%)	50 (4.2%)	28 (7.7%)	
Non‐Hispanic White	718 (46%)	545 (46%)	173 (48%)	
Other Hispanic	349 (22%)	326 (27%)	23 (6.4%)	
Other/multiracial	106 (6.8%)	82 (6.9%)	24 (6.6%)	
Education				< 0.001
9–11th grade (12th grade with no diploma)	291 (19%)	174 (15%)	117 (32%)	
College graduate or above	259 (17%)	213 (18%)	46 (13%)	
High school graduate/GED	342 (22%)	274 (23%)	68 (19%)	
Less than 9th grade	431 (28%)	352 (30%)	79 (22%)	
Some college or AA	230 (15%)	178 (15%)	52 (14%)	
Diabetes				< 0.001
No	1088 (70%)	862 (72%)	226 (62%)	
Yes	465 (30%)	329 (28%)	136 (38%)	
BMI	29.59 (7.27)	29.02 (7.30)	31.43 (6.85)	< 0.001
Drinking frequency				0.2
Non‐drinker	561 (36%)	428 (36%)	133 (37%)	
1–5 drinks/month	721 (46%)	543 (46%)	178 (49%)	
5–10 drinks/month	89 (5.7%)	75 (6.3%)	14 (3.9%)	
10+ drinks/month	182 (12%)	145 (12%)	37 (10%)	
Smoking status				< 0.001
Never	789 (51%)	604 (51%)	185 (51%)	
Former	451 (29%)	319 (27%)	132 (36%)	
Now	313 (20%)	268 (23%)	45 (12%)	
Dairy energy intake (Kcal/day)	1883.52 (898.90)	1952.06 (928.63)	1658.02 (751.46)	< 0.001
Physical activity				< 0.001
Active	232 (15%)	182 (15%)	50 (14%)	
Highly active	553 (36%)	468 (39%)	85 (23%)	
Inactive	768 (49%)	541 (45%)	227 (63%)	
Total fat mass (g)				< 0.001
Q1 (6452.8–21 120.0)	389 (25%)	338 (28%)	51 (14%)	
Q2 (21 132.0–28 024.3)	388 (25%)	305 (26%)	83 (23%)	
Q3 (28 030.0–36 385.7)	388 (25%)	261 (22%)	127 (35%)	
Q4 (36415.1–84493.4)	388 (25%)	287 (24%)	101 (28%)	
CONUT				0.7
< 3	1519 (98%)	1166 (98%)	353 (98%)	
≥ 3	34 (2.2%)	25 (2.1%)	9 (2.5%)	
CKD stage				0.07
V	19 (1.2%)	15 (1.3%)	4 (1.1%)	
IV	37 (2.4%)	24 (2.0%)	13 (3.6%)	
IIIb	127 (8.2%)	85 (7.1%)	42 (12%)	
IIIa	380 (24%)	291 (24%)	89 (25%)	
II	325 (21%)	242 (20%)	83 (23%)	
I	665 (43%)	534 (45%)	131 (36%)	
BMI category				< 0.001
Normal	435 (28%)	384 (32%)	51 (14%)	
Overweight	481 (31%)	363 (30%)	118 (33%)	
Obesity	637 (41%)	444 (37%)	193 (53%)	
HbA1C(%)	6.13 (1.63)	6.08 (1.62)	6.32 (1.65)	< 0.001
eGFR, mL/min/1.73 m^2^	80.58 (31.23)	82.24 (31.47)	75.12 (29.83)	< 0.001
LDL‐C, mmol/L	3.06 (1.02)	3.05 (1.04)	3.06 (0.92)	0.3
HDL‐C, mmol/L	1.37 (0.44)	1.40 (0.45)	1.29 (0.39)	< 0.001
TC, mmol/L	7737.56 (1746.06)	7724.79 (1787.46)	7779.55 (1603.95)	0.2
TG, mmol/L	1.63 (0.84)	1.56 (0.81)	1.85 (0.87)	< 0.001
Scr, μmol/L	95.53 (79.57)	96.29 (86.34)	93.04 (51.35)	0.8
Uric acid, μmol/L	352.16 (100.69)	347.84 (98.39)	366.36 (106.83)	0.017
Albumin, g/L	41.43 (3.72)	41.54 (3.74)	41.08 (3.62)	0.008
FPG, mmol/L	121.00 (54.95)	118.76 (52.60)	128.40 (61.55)	< 0.001
Serum potassium, mmol/L	4.09 (0.42)	4.09 (0.42)	4.11 (0.43)	0.2
Serum calcium, μmol/L	2.36 (0.11)	2.36 (0.11)	2.36 (0.10)	> 0.9
BUN, mmol/L	5.99 (3.38)	5.80 (3.16)	6.63 (3.93)	< 0.001
AST, IU/L	25.88 (19.17)	25.77 (19.37)	26.27 (18.52)	0.10
ALT, IU/L	25.65 (52.21)	25.61 (58.46)	25.78 (21.30)	0.023
TBil, μmol/L	12.17 (7.39)	12.19 (8.00)	12.09 (4.88)	0.5
SBP, mmHg	137.71 (25.92)	135.46 (25.73)	145.09 (25.21)	< 0.001
DBP, mmHg	70.87 (17.89)	71.00 (17.62)	70.43 (18.75)	> 0.9

Abbreviations: BMI, body mass index; HbA1C, haemoglobin A1c; eGFR, estimated glomerular filtration rate; LDL‐C, low‐density lipoprotein cholesterol; HDL‐C, high‐density lipoprotein cholesterol; TC, total cholesterol; TG, triglycerides, LDH, lactate dehydrogenase; FPG, fasting plasma glucose; Scr, serum creatinine; BUN, blood urea nitrogen; AST, aspartate transaminase; ALT, alanine aminotransferase; TBil, total bilirubin; SBP, systolic blood pressure; DBP, diastolic blood pressure.

^a^ Median (IQR) for continuous; *n* (%) for categorical.

^b^ Wilcoxon rank sum test.

### 3.2 Association Between Sarcopenia and Mortality of Adults With CKD

Sarcopenia was positively associated with all‐cause mortality risk in the crude model (hazard ratio [HR] 1.64; 95% CI 1.40–1.93, *p* < 0.001) (Table 2). After adjusting for age, sex, race, BMI, systolic blood pressure, smoking and drinking status, co‐diabetes, MET level, daily energy intake, CONUT group, total fat mass group and laboratory indicators, all‐cause mortality remained significantly higher in the sarcopenia population (HR 1.21, 95% CI 1.00–1.45, *p* = 0.047).

**TABLE 2:** The association between sarcopenia and the risk of all‐cause mortality.

	HR (95% CI) *p*	
	Non‐sarcopenia	Sarcopenia
All‐cause mortality		
Model 1	Reference	1.64 (1.40–1.93) *p* < 0.001
Model 2	Reference	1.20 (1.01–1.42) *p* = 0.043
Model 3	Reference	1.21 (1.00–1.45) *p* = 0.047

*Note:* Model 1: no covariates were adjusted. Model 2: age, gender, race, education, smoking status, drinking frequency and diabetes were adjusted. Model 3: gender, age, race, education, smoking status, drinking frequency, diabetes, MET level, daily energy intake, CONUT group, total fat mass quartiles, eGFR levels, systolic blood pressure, serum potassium, serum calcium, uric acid levels and lipid metabolism indicators (TC, TG, LDL and HDL) were adjusted.

Abbreviations: 95% CI, 95% confidence interval; HR, hazard ratio.

### 3.3 Association Between Obesity/SO With Mortality of Adults With CKD

We classified the population into normal and obesity groups based on BMI. The crude model showed that the obesity group had a lower risk for all‐cause mortality (HR 0.66, 95% CI 0.56–0.77, *p* < 0.001). However, after adjusting for covariates, the HR for mortality outcomes was 0.96 (95% CI 0.80–1.15, *p* = 0.628) (Table 3).

**TABLE 3:** The association between obesity and the risk of all‐cause mortality.

	HR (95% CI) *p*	
	Non‐obesity	Obesity
All‐cause mortality		
Model 1	Reference	0.66 (0.56–0.77) *p* < 0.001
Model 2	Reference	0.99 (0.83–1.18) *p* = 0.912
Model 3	Reference	0.96 (0.80–1.15) *p* = 0.628

*Note:* Model 1: no covariates were adjusted. Model 2: age, gender, race, education, smoking status, drinking frequency and diabetes were adjusted. Model 3: gender, age, race, education, smoking status, drinking frequency, diabetes, sarcopenia, MET level, daily energy intake, CONUT group, total fat mass quartiles, CKD stage, systolic blood pressure, serum potassium, serum calcium, uric acid levels and lipid metabolism indicators (TC, TG, LDL and HDL) were adjusted.

Abbreviations: 95% CI, 95% confidence interval; HR, hazard ratio.

Next, we divided all CKD participants into four groups based on BMI and sarcopenia index: the only sarcopenia group, the normal group, the SO group and the only obesity group. As shown in Table 4, in Model 1, compared with the normal group, the sarcopenia group had a significantly increased all‐cause mortality risk (HR 1.77, 95% CI 1.44–2.19, *p* < 0.001) whereas the obesity group showed a significant reduction in all‐cause mortality risk (HR 0.59, 95% CI 0.48–0.72, *p* < 0.001). No significant difference was observed in the SO group. After multivariable adjustment, the sarcopenia group still showed an increased mortality risk (HR 1.26, 95% CI 1.01–1.58, *p* = 0.044), but no difference was found in the obesity and SO groups.

**TABLE 4:** The association between sarcopenia/obesity and the risk of all‐cause mortality.

HR (95% CI) *p*				
All‐cause mortality	Normal (*N* = 748)	Only sarcopenia (*N* = 173)	Only obesity (*N* = 443)	Sarcopenic obesity (*N* = 189)
Model 1	Reference	1.77 (1.44–2.19) *p* < 0.001	0.59 (0.48–0.72) *p* < 0.001	1.10 (0.88–1.38) *p* = 0.390
Model 2	Reference	1.20 (0.96–1.50) *p* = 0.103	0.94 (0.76–1.17) *p* = 0.585	1.16 (0.91–1.46) *p* = 0.227
Model 3	Reference	1.26 (1.01–1.58) *p* = 0.044	1.02 (0.76–1.38) *p* = 0.886	1.30 (0.96–1.77) *p* = 0.094

*Note:* Model 1: no covariates were adjusted. Model 2: age, gender, race, education, smoking status, drinking frequency and diabetes were adjusted. Model 3: gender, age, race, education, smoking status, drinking frequency, diabetes, MET level, daily energy intake, CONUT group, total fat mass quartiles, eGFR levels, systolic blood pressure, serum potassium, serum calcium, uric acid levels and lipid metabolism indicators (TC, TG, LDL and HDL) were adjusted.

Abbreviations: 95% CI, 95% confidence interval; HR, hazard ratio.

Stratified analysis based on the severity of CKD and the presence of diabetes revealed that obesity had a protective effect on mortality risk in CKD Stages I–II (HR 0.45, 95% CI 0.28–0.72, *p* = 0.001) (Table 5), but became a mortality risk factor in CKD Stages III–V (CKD III: HR 1.67, 95% CI 1.07–2.60, *p* = 0.024; CKD IV–V: HR 17.51, 95% CI 1.29–238.01, *p* = 0.032). Sarcopenia significantly increased mortality risk in patients without diabetes (HR 1.40, 95% CI 1.07–1.84, *p* = 0.016), whereas SO only posed a mortality risk in advanced CKD patients (HR 106.49, 95% CI 8.60–1318.45, *p* < 0.001) (Table 5).

**TABLE 5:** The association between sarcopenia/obesity and the risk of all‐cause mortality.

HR (95% CI) *p*				
All‐cause mortality	Normal	Only sarcopenia	Only obesity	Sarcopenic obesity
CKD I–II	Reference	1.03 (0.71–1.49) *p* = 0.889	0.45 (0.28–0.72) *p* = 0.001	0.71 (0.45–1.14) *p* = 0.157
CKD III	Reference	1.21 (0.88–1.66) *p* = 0.250	1.67 (1.07–2.60) *p* = 0.024	1.38 (0.87–2.18) *p* = 0.170
CKD IV–V	Reference	4.01 (0.50–32.31) *p* = 0.191	17.51 (1.29–238.01) *p* = 0.032	106.49 (8.60–1318.45) *p* < 0.001
Without diabetes	Reference	1.40 (1.07–1.84) *p* = 0.016	1.00 (0.66–1.52) *p* = 0.981	1.24 (0.84–1.82) *p* = 0.285
With diabetes	Reference	1.09 (0.69–1.72) *p* = 0.703	0.82 (0.49–1.36) *p* = 0.443	1.19 (0.68–2.11) *p* = 0.540

*Note:* Model 1: no covariates were adjusted. Model 2: age, gender, race, education, smoking status, drinking frequency and diabetes were adjusted. Model 3: gender, age, race, education, smoking status, drinking frequency, diabetes, MET level, daily energy intake, CONUT group, total fat mass quartiles, eGFR levels, systolic blood pressure, serum potassium, serum calcium, uric acid levels and lipid metabolism indicators (TC, TG, LDL and HDL) were adjusted.

Abbreviations: 95% CI, 95% confidence interval; HR, hazard ratio.

### 3.4 Association Between fat Mass With Mortality of Adults With CKD

Considering that BMI cannot differentiate specific body compositions, we categorized participants into quartiles based on total fat mass. The results showed that after adjusting for various covariates, the Q2 group had a protective effect compared with Q1 (HR 0.78, 95% CI 0.62–0.99, *p* = 0.039) (Table 6). For patients with Stage 3 CKD, it appears that greater fat mass may provide more benefits (Q2: HR 0.64, 95% CI 0.47–0.88, *p* = 0.006; Q3: HR 0.62, 95% CI 0.41–0.93, *p* = 0.021). Further subgroup analysis by sex revealed that in the male population, fat mass in the Q2 (HR 0.71, 95% CI 0.51–0.99, *p* = 0.046) and Q3 (HR 0.62, 95% CI 0.41–0.92, *p* = 0.019) quartiles was associated with a reduced mortality risk, whereas no protective effect of fat mass was observed in the female population (Table 6).

**TABLE 6:** Total fat mass quartiles and all‐cause mortality in CKD patients.

HR (95% CI) *p*				
All‐cause mortality	Q1	Q2	Q3	Q4
All	Reference	0.78 (0.62–0.99) *p* = 0.039	0.83 (0.62–1.11) *p* = 0.206	0.93 (0.59–1.45) *p* = 0.742
Sarcopenia	Reference	0.59 (0.35–0.99) *p* = 0.045	0.57 (0.31–1.05) *p* = 0.071	0.61 (0.25–1.48) *p* = 0.271
Non‐ sarcopenia	Reference	0.78 (0.59–1.02) *p* = 0.069	0.83 (0.59–1.18) *p* = 0.305	0.96 (0.56–1.66) *p* = 0.893
CKD stages				
CKD I–II	Reference	0.92 (0.64–1.33) *p* = 0.675	1.12 (0.72–1.73) *p* = 0.610	1.49 (0.75–2.94) *p* = 0.254
CKD III	Reference	0.64 (0.47–0.88) *p* = 0.006	0.62 (0.41–0.93) *p* = 0.021	0.58 (0.31–1.09) *p* = 0.092
CKD IV–V	Reference	0.52 (0.14–1.91) *p* = 0.321	4.49 (0.59–34.09) *p* = 0.146	14.86 (0.76–289.01) *p* = 0.075
Male				
All	Reference	0.71 (0.51–0.99) *p* = 0.046	0.62 (0.41–0.92) *p* = 0.019	0.86 (0.48–1.53) *p* = 0.607
Sarcopenia	Reference	1.11 (0.52–2.35) *p* = 0.796	0.47 (0.22–0.97) *p* = 0.042	0.68 (0.26–1.75) *p* = 0.422
Non‐ sarcopenia	Reference	0.57 (0.37–0.86) *p* = 0.008	0.57 (0.34–0.95) *p* = 0.032	0.78 (0.39–1.58) *p* = 0.491
Female				
All	Reference	0.73 (0.51–1.03) *p* = 0.073	0.70 (0.44–1.09) *p* = 0.110	0.75 (0.36–1.54) *p* = 0.433
Sarcopenia	Reference	0.36 (0.11–1.17) *p* = 0.091	0.34 (0.09–1.25) *p* = 0.104	0.38 (0.04–3.56) *p* = 0.396
Non‐ sarcopenia	Reference	0.90 (0.60–1.33) *p* = 0.588	0.77 (0.47–1.27) *p* = 0.302	0.88 (0.38–2.03) *p* = 0.764

*Note:* Adjusted covariates: gender, age, race, education, smoking status, drinking frequency, diabetes, MET level, daily energy intake, CONUT group, BMI, eGFR levels, systolic blood pressure, serum potassium, serum calcium, uric acid levels and lipid metabolism indicators (TC, TG, LDL and HDL).

Abbreviations: 95% CI, 95% confidence interval; HR, hazard ratio.

### 3.5 Subgroup Analysis

**FIGURE 1:**
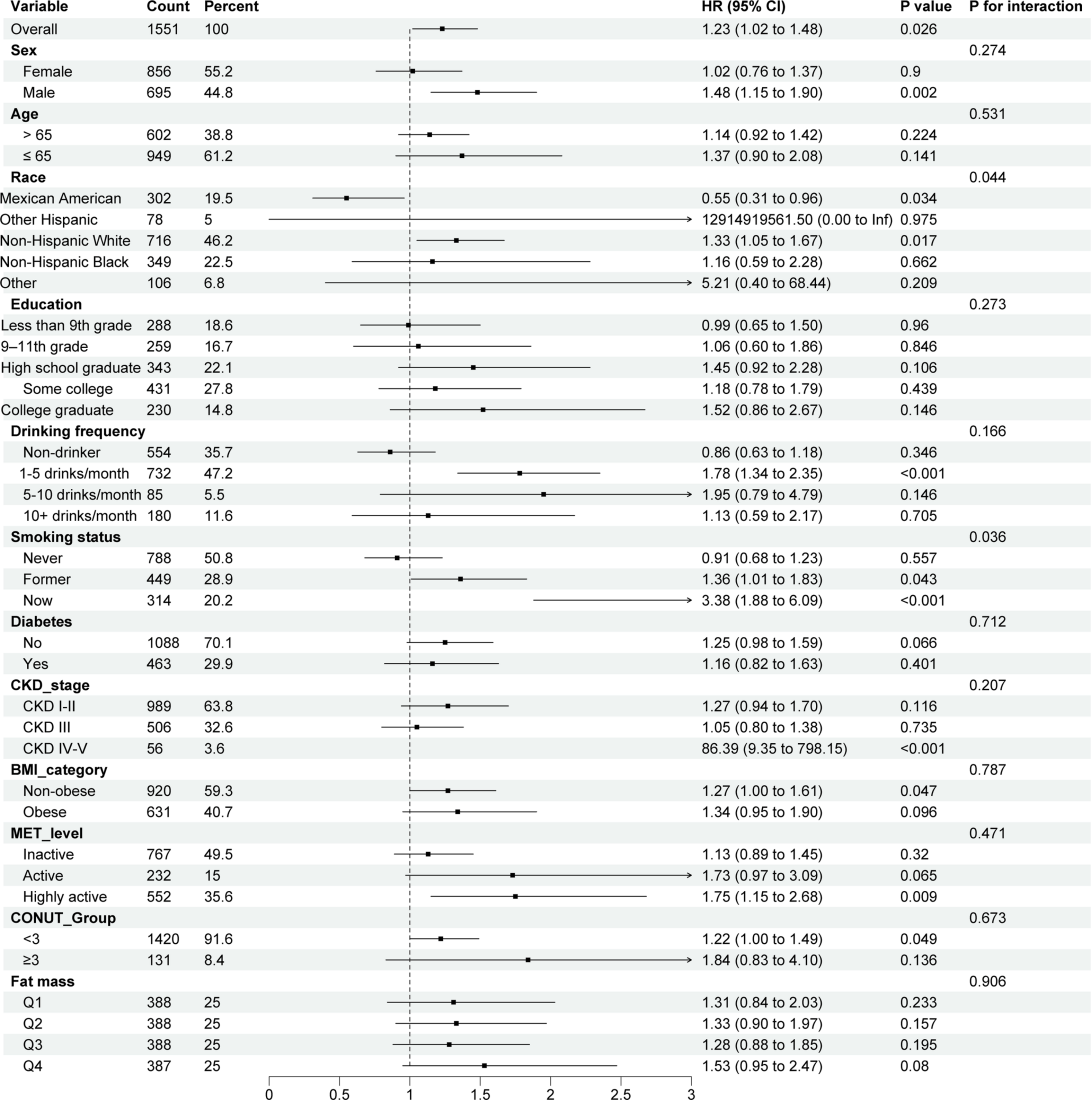
Subgroup analysis for the association between the sarcopenia and the risk of all‐cause mortality.

In our subgroup analysis and interaction tests, we examined the relationship between sarcopenia and all‐cause mortality risk across different population subgroups (gender, age, race, education, smoking and drinking status, diabetes status, CKD stage, obesity status, nutritional status and PA level) (Figure 1). The risk of mortality was increased in males (HR 1.48, 95% CI 1.15–1.90, *p* = 0.002), non‐Hispanic Whites (HR 1.33, 95% CI 1.05–1.67, *p* = 0.017), those drinking 1–5 times per month (HR 1.78, 95% CI 1.34–2.35, *p* < 0.001), past smokers (HR 1.36, 95% CI 1.01–1.83, *p* = 0.043), current smokers (HR 3.38, 95% CI 1.88–6.09, *p* < 0.001), non‐obese individuals (HR 1.27, 95% CI 1.00–1.61, *p* = 0.047), those with CKD Stages IV–V (HR 86.4, 95% CI 9.35–798.15, *p* = 0.008) and individuals with high PA (HR 1.75, 95% CI 1.15–2.68, *p* = 0.009). No other interaction factors were observed except for race (*p* = 0.044).and smoking status (*p* = 0.036).

### 3.6 RCS Regression Analysis

**FIGURE 2:**
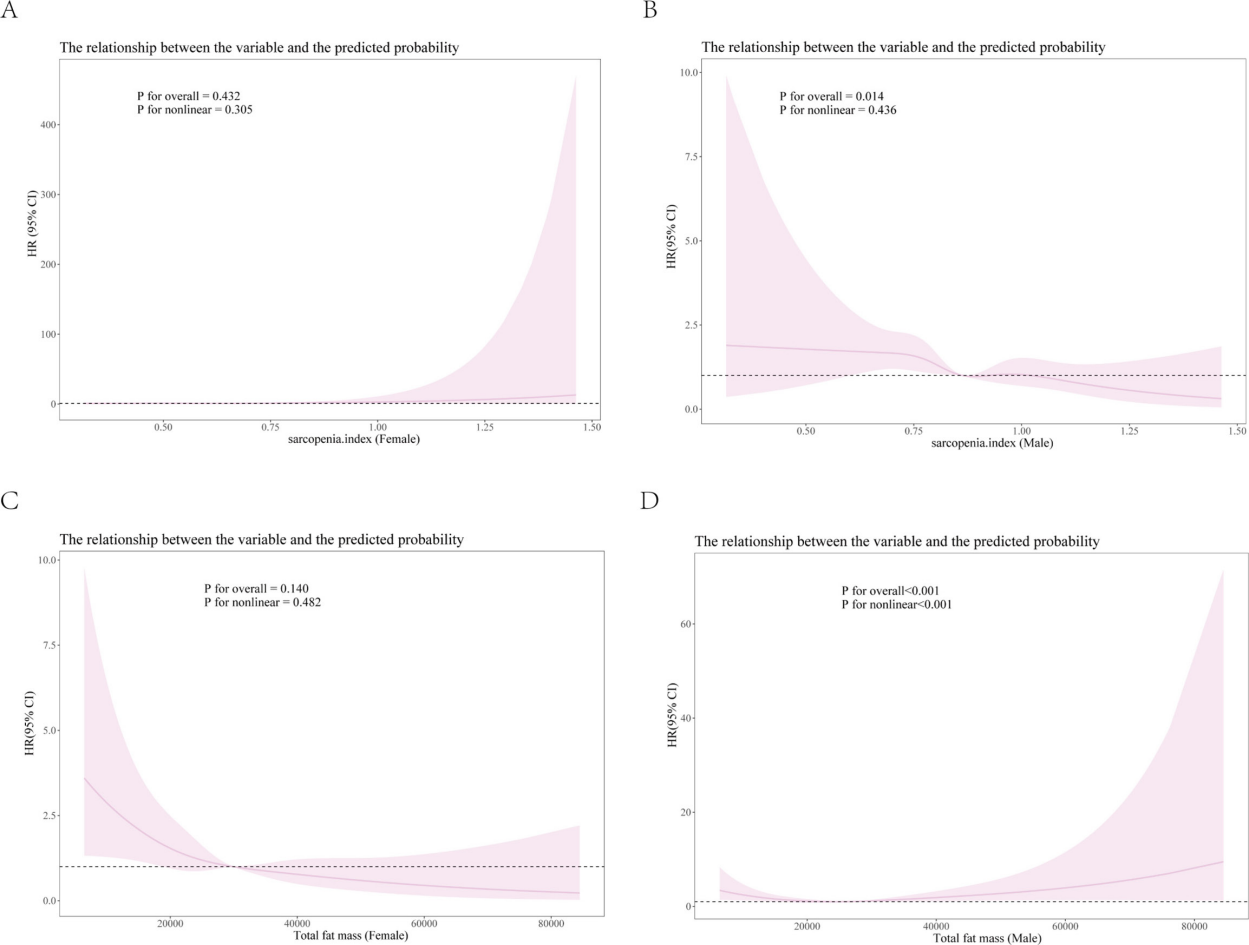
Restricted cubic spline curve for the association of sarcopenia index and fat mass with mortality after adjusting for all variables. Association between (A) sarcopenia index and all‐cause mortality in the female population, (B) sarcopenia index and all‐cause mortality in the male population, (C) fat mass and all‐cause mortality in the female population and (D) fat mass and all‐cause mortality in the male population.

RCS and Cox proportional hazards regression models were used to assess the relationship between the sarcopenia index and all‐cause mortality. Figure 2A shows that, after adjusting for all covariates, the sarcopenia index was not significantly associated with all‐cause mortality in the female population (*p* for overall = 0.432, *p* for nonlinear = 0.305). In the male population, after adjusting for all covariates, there was a linear relationship between the sarcopenia index and all‐cause mortality, and the association was significant (*p* for overall = 0.014, *p* for nonlinear = 0.436). The first inflection point was at 0.86, where, to the left of the inflection point, the all‐cause mortality risk gradually decreased as the sarcopenia index increased (Figure 2B).

We also used RCS and Cox proportional hazards regression models to assess the relationship between fat mass and all‐cause mortality. Figure 2C shows that, after adjusting for all covariates, fat mass was not significantly associated with all‐cause mortality in the female population (*p* for overall = 0.140, *p* for nonlinear = 0.482). In the male population, after adjusting for all covariates, there was a nonlinear relationship between fat mass and all‐cause mortality, and the association was significant (*p* for overall < 0.001, *p* for nonlinear < 0.001). To the left of the inflection point (fat mass = 21 257 g), all‐cause mortality risk gradually decreased with increasing fat mass. To the right of the inflection point (fat mass = 28 104 g), the all‐cause mortality risk gradually increased with increasing fat mass (Figure 2D).

## 4 Discussion

In a nationally representative sample of US adults, we found that sarcopenia increases the risk of all‐cause mortality in CKD patients. The impact of obesity, as defined by BMI, on mortality risk appears to vary across different stages of CKD. Gender‐based subgroup analyses indicated that moderate to higher levels of fat mass seem to benefit survival in male CKD patients and those in Stage 3 CKD. Sensitivity analyses confirmed the robustness of these findings, with stronger associations observed in certain subgroups.

Recent studies have also investigated the relationship between sarcopenia and prognosis in CKD patients. Wu et al. found that sarcopenia and mild kidney dysfunction synergistically increase the risk of all‐cause and specific‐cause mortality. Early detection and improvement of sarcopenia may reduce mortality risk [26]. A Japanese team also discovered that sarcopenia was associated with poor survival and kidney outcomes in older patients with CKD [27]. Meta‐analyses have suggested that sarcopenia is an important predictor of cardiovascular events and mortality among dialysis patients [28]. These findings are consistent with our study's conclusion that sarcopenia is an independent risk factor for mortality in CKD patients. Additionally, we found that the risk of sarcopenia was more pronounced in non‐obese, non‐diabetic men, possibly due to the lack of sufficient fat reserves in these individuals to compensate for the negative effects of reduced muscle mass [29].

There are several possible explanations for the association between sarcopenia and mortality in CKD patients. Malnutrition and insufficient energy reserves, chronic inflammation and metabolic disturbances in CKD patients contribute to increased mortality risk [30]. Sarcopenia exacerbates muscle loss, reducing energy reserves and compensatory capacity during acute events. Persistent low‐grade inflammation (e.g., elevated IL‐6 and TNF‐α) and metabolic abnormalities (e.g., protein catabolism and acidosis) accelerate muscle breakdown and impair survival [31]. Additionally, sarcopenia leads to reduced muscle strength and PA, heightening fall risks and complications while worsening metabolic dysfunction in a vicious cycle [32]. Furthermore, sarcopenia amplifies cardiovascular risks, with studies linking muscle loss to higher cardiovascular event rates through metabolic, vascular and inflammatory pathways [33].

Obesity is a risk factor for mortality in CKD patients, and sarcopenia also increases mortality risk in this population. However, whether SO further exacerbates all‐cause mortality in CKD patients remains controversial [34, 35]. Previous studies suggest that nutritional differences and body composition may explain the observed survival advantages in obese patients with end‐stage kidney disease (ESKD) [36]. Additionally, research has shown that weight loss in obese adult CKD patients is associated with higher mortality rates (Management of Patients With Kidney Disease Undergoing Bariatric Surgery: A Multidisciplinary Approach) [2, 37].

Our findings also indicate that higher BMI may be beneficial for survival in early‐stage CKD patients but becomes a significant risk factor in the middle‐to‐late stages of CKD. Because BMI does not distinguish between muscle and fat mass, we further analysed fat mass and found that moderate or higher fat mass, compared with low fat mass, provides a survival advantage for Stage 3 CKD patients and male populations, resembling the commonly described ‘obesity paradox’ [38, 39, 40]. Interestingly, using RCS analysis to evaluate the trend changes in overall mortality risk based on the continuous variables sarcopenia index and fat mass, we found significant associations between these variables and mortality risk in male patients after adjusting for covariates such as age, sex, race, education level, diabetes status, BMI, CKD stage, PA and nutritional status. Specifically, a sarcopenia index below 0.86 and total fat mass in the range of 21 257–28 104 g were associated with a potential reduction in mortality risk. Some studies suggest that weight loss may improve the prognosis of CKD patients. However, based on our findings, rapid weight loss methods, including surgery or strict dieting, are not recommended. Instead, focusing on moderate muscle‐building strategies may be more beneficial for improving survival outcomes in CKD patients [27, 41].

This study has several strengths. First, it included patient data up to 2018 to evaluate the mortality risk associated with sarcopenia and BMI‐defined obesity in CKD patients and explored the impact of fat mass on survival across different CKD stages and genders. However, the study has several limitations. The primary limitation of this study is the measurement of body composition at a single time point. Therefore, we cannot infer causality between mortality rates and changes in body composition. Because over time, dynamic changes in body composition, such as muscle and fat, may exacerbate or mitigate the relationship between CKD and mortality risk. Additionally, this study excluded individuals lacking critical data, including information on diabetes diagnosis, blood lipids and drinking or smoking questionnaire responses, as these factors may impact the interpretation of the results. Future large‐scale studies are needed to further explore these associations. Excessive extracellular fluid (such as oedema in CKD patients) can lead to overestimation of muscle (lean tissue) mass by DXA, which may affect the association between sarcopenia and mortality in CKD patients [42]. Lastly, our study used the low appendicular muscle mass thresholds recommended by the National Institutes of Health Foundation (FNIH) to define sarcopenia [23]. Although current guidelines also recommend including functional measurements in the definition, we focused on muscle mass due to the limited number of individuals in the NHANES cohort who underwent functional assessments. Despite these limitations, our study incorporated energy intake, PA levels and nutritional status and still identified an association between low muscle mass and the mortality risk in CKD patients.

## 5 Conclusion

Sarcopenia is an independent risk factor for all‐cause mortality in CKD patients. In male CKD patients and those in Stage 3 CKD, moderate to higher fat mass may confer survival benefits. Future prospective studies are needed to further analyse the relationship between muscle and fat mass and survival in CKD patients.

## Author Contributions

Conceptualization, J.E.L.; data curation, H.T., Y.Y. Z. and S.Q.Y.; investigation, H.T.; methodology and writing original draft, J.E.L.; review and revise, J.P.L. and P.Y. All authors have read and agreed to the published version of the manuscript.

## Ethics Statement

This research followed the STROBE reporting guideline. The studies involving human participants were reviewed and approved by the NCHS Research Ethics Review Board (ERB) (Protocol #98–12, #2006–06, #2011–17 and #2018–01, https://www.cdc.gov/nchs/nhanes/irba98.htm).

## Consent

All participants signed the informed consent before participating in the study. All methods were carried out in accordance with relevant guidelines and regulations. All the authors agreed to submit the manuscript to this journal for consideration.

## Conflicts of Interest

The authors declare no conflicts of interest.

## Data Availability

The data of National Health and Nutrition Examination Survey (NHANES) can be downloaded online (https://www.cdc.gov/nchs/nhanes/index.html).
